# Huang-Lian-Jie-Du-Decoction Ameliorates Hyperglycemia and Insulin Resistant in Association With Gut Microbiota Modulation

**DOI:** 10.3389/fmicb.2018.02380

**Published:** 2018-10-08

**Authors:** Mingyi Chen, Ziqiong Liao, Biyu Lu, Mengxia Wang, Lei Lin, Shaobao Zhang, Yuan Li, Deliang Liu, Qiongfeng Liao, Zhiyong Xie

**Affiliations:** ^1^School of Pharmaceutical Sciences, Sun Yat-sen University, Guangzhou, China; ^2^School of Pharmaceutical Sciences, Guangzhou University of Chinese Medicine, Guangzhou, China; ^3^School of Pharmaceutical Sciences (Shenzhen), Sun Yat-sen University, Guangzhou, China

**Keywords:** 16S gene sequencing, gut microbiota, HLJDD, metagenomics, type 2 diabetes mellitus

## Abstract

**Background:** Huang-Lian-Jie-Du-Decoction (HLJDD), a prescription of traditional Chinese medicine, has been clinically used to treat diabetes for thousands of years and its mechanism was reported to be related to gut microbiota. However, no study has explored the effect of HLJDD on the gut microbiota in type 2 diabetes mellitus (T2DM) yet. Therefore, in this study, we investigated the modulation of gut microbiota induced by HLJDD treatment in T2DM in order to unveil the underlying mechanism.

**Methods:** A combination of high-fat diet (HFD) and streptozotocin (STZ) was used to induce T2DM in rats. Bacterial communities in the fecal samples from the control group, the T2DM model group, and the HLJDD-treated T2DM group were analyzed by 16S gene sequencing, followed with a subset sample analyzed by shotgun sequencing.

**Results:** The HLJDD treatment significantly ameliorated hyperglycemia and inflammation in T2DM rats. Additionally, our results indicated that HLJDD treatment could not only restore the gut dysbiosis in T2DM rats, which was proved by an increasing amount of short chain fatty acids (SCFAs)-producing and anti-inflammatory bacteria such as *Parabacteroides*, *Blautia*, and *Akkermansia* as well as a decreasing amount of conditioned pathogenic bacteria (e.g., *Aerococcus*, *Staphylococcus*, and *Corynebacterium*), but also modulate the dysregulated function of gut microbiome in T2DM rats, including an up-regulation in bile acid biosynthesis as well as a reduction in glycolysis/gluconeogenesis and nucleotide metabolism.

**Conclusion:** HLJDD treatment could ameliorate hyperglycemia and restore the dysregulated microbiota structure and function to a normal condition mainly by increasing SCFAs-producing bacteria and reducing conditioned pathogenic bacteria in T2DM rats, which provides insights into the mechanism of HLJDD treatment for T2DM from the view of gut microbiota.

## Introduction

Huang-Lian-Jie-Du-Decoction, a famous prescription of TCM, is first recorded in the book “Wai-Tai-Mi-Yao” in the Tang dynasty. It has been clinically practiced in the treatment of T2DM in China and officially listed in the Chinese Pharmacopoeia (Zhang et al., 2014). HLJJDD consists of four herbs including Huanglian (Coptidis Rhizoma), Huangqin (Scutellariae Radix), Huangbo (Phellodendri Cortex), and Zhizi (Gardeniae Fructus). As the previous studies reported, HLJDD achieved great therapeutic effects in ameliorating cerebral ischemia (Wang et al., 2013), anti-inflammation (Zeng et al., 2009), inhibiting the lipid peroxidation (Ikarashi et al., 2012), and modulating the lipid metabolism (Jin et al., 2010; Zhang et al., 2014). Except for the extensive use of HLJDD in ancient China, recent studies indicated that both HLJDD and some components of HLJDD could lower the levels of plasma glucose and improve insulin resistance via several different pathways (Zhang et al., 2014). For example, berberine, a quaternary ammonium alkaloid isolated from *Rhizoma Coptidis*, ameliorated insulin resistance through suppressing the activation of M1 macrophage (classically activated macrophage) in adipose tissue (Ye et al., 2016). Baicalin, one of the key components in Scutellariae Radix, demonstrated antioxidant properties and hypoglycemic activity in diabetic rats (Waisundara et al., 2009). Geniposide, one of the components in Gardeniae Fructus, was reported to regulate glucose-stimulated insulin secretion through controlling glucose metabolism (Liu et al., 2013). From the studies mentioned above, it is suggested that the treating modality of HLJDD is a systemic treatment that has multi-components, multi-paths, multi-targets, and multi-effects (Zhang et al., 2014), functioning as a comprehensive treatment of T2DM and its complications. However, the underlying mechanisms of HLJDD treatment in ameliorating T2DM are still not fully understood.

Recently, evidence has accumulated that the development of T2DM and the mechanism of its therapeutic drugs may be related to the gut microbiome (Delzenne et al., 2015; Holmes, 2016; Brunkwall and Orho-Melander, 2017). Gut microbiota can produce many substances that could induce inflammation in peripheral tissues and cause insulin resistance and T2DM in the body when they enter the blood from the gut lumen, such as peptidoglycan and LPS (Wei et al., 2018). Hence, it is a potential way to illuminate the mechanism of HLJDD from the perspective of gut microbiota as considering the key role of microbiota in health maintenance. Actually, a series of studies have been performed to investigate the therapeutic effects of some monomers in HLJDD from the point of gut microbiota. For instance, it is reported that oral administration of berberine could modify the mice gut microbiota composition and increase the production of butyrate, furthering lower the glycolipid levels when it enters blood (Wang et al., 2017). Han et al. (2017) showed that combination treatment with metformin, a classic antidiabetic drug, and Scutellariae Radix could improve obesity-induced insulin resistance and lipid metabolism by maintaining bile acid homeostasis through reducing the amount of harmful bacteria and altering the gut microbiota structure. From the perspective of holistic view of TCM, different herbs and multiple components in HLJDD could act on multiple targets and exert an enhancing synergistic effect (Fang et al., 2013). Thus, the results based on the previous studies are partial and indirect. Further investigations are in great need to clarify the effects of HLJDD on gut microbiota. However, no study assessed the impact of HLJDD treatment on the gut microbiota niche in disease states like T2DM, which prompted us to explore the effects of HLJDD on gut microbiome to elaborate the mechanism in alleviating T2DM.

As mentioned above, the mechanism of HLJDD in the treatment of T2DM is suggested to be correlated with gut microbiome which is still not fully understood. Therefore, the present study aimed to investigate the effects of HLJDD treatment on the composition and function of gut microbiota in HFD combined with STZ-induced T2DM rats by using 16S rRNA gene sequencing and shotgun metagenomic sequencing. Moreover, correlation analysis between T2DM-related metabolic factors and gut microbiota was conducted to identify the key bacteria in ameliorating T2DM. We intended to investigate the mechanisms of HLJDD in treating T2DM from the perspective of gut microbiome, which may help elucidate the possible mechanisms of the protective effects against T2DM brought by HLJDD.

## Materials and Methods

### Preparation of Huang-Lian-Jie-Du-Decoction

The TCM formula in our study was HLJDD, composed of four herbs, namely: Huanglian (Coptidis Rhizoma), Huangqin (Scutellariae Radix), Huangbo (Phellodendri Cortex), and Zhizi (Gardeniae Fructus). Herbs were all provided and their quality was controlled by Zisun Chinese Pharm Co. Ltd. (Guangzhou, China). We prepared HLJDD referred to the optimized method described by Zhang et al. (2014). Details are as follows: distilled water was used for the extraction and preparation of samples. Coptidis Rhizoma, Scutellariae Radix, Phellodendri Cortex, and Gardeniae Fructus were crushed into small pieces and mixed in a ratio of 3:2:2:3 (weight). The mixture was refluxed with water (1:10, w/v) for 2 h. Filtrates were collected and the residues was then refluxed in water (1:10, w/v) for 1.5 h. The two filtrates were combined and concentrated. Afterward, the concentrated extract was dried by vacuum concentration and the HLJDD extract was obtained at a yield of 22.8% (w/w, dried extract/crude herb). The extract was grinded into powder and stored in seal at 4°C. The freeze-dried powder was suspended in 0.5% CMC-Na solution when used in the experiment.

### Quantitative Analysis of Marker Components of HLJDD Extract

The contents of marker components contained in HLJDD extract were analyzed with HPLC method; 10 mL methanol was added to 100 mg extract power and the mixture was put under vortex movement for 5 min. After centrifugation at 12,000 rpm for 30 min, the supernatant was analyzed using a HPLC method. HPLC was performed on a Shimadzu LC-20AT HPLC system equipped with a LC workstation CBM-10A VP Plus (Shimadzu, Japan). The assays of geniposide, baicalin, and berberine were conducted according to the reported methods (Huang et al., 2011) with a minor modification. Briefly, acetonitrile (A) and 0. 05% potassium dihydrogen phosphate mixed with 0. 05% triethanolamine (pH was regulated to 3.0 by phosphoric acid) (B) were used as mobile phase at a flow rate of 1.0 mL⋅min^-1^. The samples were separated by a reversed-phase HPLC column (JADE-PAK ODS-AQ C18 250 mm × 4.6 mm I.D. 5 μm particle size) with a linear gradient elution under predetermined procedures (0–9 min, 83% B; 9–20 min, 83%→72% B; 20–28 min, 72%→63% B; 33–42 min, 63%→55% B; 40–45 min, 55%→83% B). The solvent was filtered through a 0.45 μm Millipore filter and degassed prior to use. The injection volume was 20 μL and the operating temperature was maintained at 25°C. Ultraviolet detection with the wavelength at 245 nm was used for berberine, baicalin, and geniposide.

### Induction of Type 2 Diabetic Rats

Pathogen-free male Sprague–Dawley rats (body weight 130 ± 20g) were supplied by the medical laboratory animal center of Guangdong province (Guangzhou, China) and housed in a specific pathogen-free animal laboratory (12 h light/dark cycle, 20°C, 50–70% humidity) with free access to food and water. All animal experimental procedures were performed at the Sun Yat-sen University Animal Experiment Center (Guangzhou, China). The protocol was reviewed and approved by the IACUC of Sun Yat-sen University and conformed to the National Institute of Health guidelines on the ethical use of animals. All efforts were made to ameliorate animals’ suffering.

After acclimatization for 7 days, a total of 32 rats were randomly divided into two groups that labeled as the NCG (*n* = 8) and HFD group (*n* = 24). The composition of the diets is shown in **Supplementary Table S1**; 4 weeks later, the 24 rats fed with HFD were fasted overnight (12 h) and received a single intraperitoneal injection of STZ [Sigma Aldrich Ltd., dissolved with 0.1 M citric acid-sodium citrate buffer (pH = 4.2–4.5) at dose of 35 mg/kg (bw); Wei et al., 2018]. Rats in the NCG group were injected with the buffer solution only. Two weeks after the injection of STZ, 16 rats whose FBG levels exceeded 11.1 mmol/L were confirmed to the T2DM model (Lu et al., 2016).

### Animal Experiment

As shown in the flow chart of the experiment (**Supplementary Figure S1**), the T2DM rats (*n* = 16) were randomly divided into two groups (*n* = 8 each group): DMG, type 2 diabetic treated with HLJDD extract (HLJDD). Rats in HLJDD group were given HLJDD extract at the dose of 1.5 g/kg suspended in 0.5% CMC-Na solution by intragastric administration for 4 weeks. Rats in NCG and DMG were given an equal volume of 0.5% CMC-Na solution. After 4 weeks of treatment, feces samples were collected (from 8:00 am to 16:00 pm) using metabolic cages with ice-packed Eppendorf tubes and immediately stored at -80°C until analysis. And at the end of the trial, all of the animals were anesthetized after an overnight fast. Serum samples were collected from the orbital plexus while pancreas, liver, and kidney tissues were harvested for further analysis.

### Oral Glucose Tolerance Test

Oral glucose tolerance test is not only used to diagnose diabetes or prediabetes, but a classical and model-based estimate of beta-cell function (Rijkelijkhuizen et al., 2009). For OGTT, the animals were subjected to overnight (12 h) fasting and administered sterilized glucose solution (2 g/kg, Sigma–Aldrich, United States) by oral gavage. The glucose levels of the blood samples collected from the tail vein were measured at four different time points (0, 0.5, 1, and 2 h) using a glucose-meter (ONETOUCH Ultra, LifeScan, United States), and the AUC during the OGTT was calculated.

### Biomedical Analysis

Body weight of all animals was monitored throughout the experiment. FBG levels of rats were determined by a glucose-meter (ONETOUCH Ultra, LifeScan, United States). Clinical chemistry analyses, including HDL-C, LDL-C, TC, TG, AST, ALT, TBA, TBIL, and DBIL, were conducted with the standard routine procedures on a Beckman CX5 automatic biochemical analyzer (Beckman Coulter, Inc., United States) in the clinical laboratory of Sun Yat-sen University Animal Experiment Center. IL-1β and IL-6 levels were evaluated by Bio-Plex^®^ multiplex immunoassays and kit (Bio-Rad, United States). MDA, SOD, GSH-Px, CRP, and FINS levels were evaluated by commercial rat ELISA kit (Jiancheng Systems China Co. Ltd., Nanjing, China). Insulin resistance index (HOMA-IR) was calculated using the following formula: HOMA-IR = FINS (mU/L) × FBG (mmol/L)/22.5.

The pancreas, liver, and kidney from rats in three groups were weighed up and the organ indexes were calculated using the following formulas: organ index (mg/g) = organ weight (mg)/body weight (g). For histopathological assessments, the kidney and pancreas were embedded in paraffin wax according to routine procedures (Rubin and Baur, 1983) after fixation for 48 h by 10% neutral-buffered formalin; 4 μm thick sections were cut and stained with hematoxylin–eosin. Examinations were performed out blindly by an experienced pathologist of Sun Yat-sen University Animal Experiment Center under microscopy.

### DNA Extraction and Polymerase Chain Reaction Amplification

Bacterial DNA was extracted from fecal samples using the MOBIO PowerSoil^®^ DNA Isolation Kit (MOBIO, United States) according to the recommendation of the manufacturer. DNA integrity and quality were evaluated by agarose gel electrophoresis (concentration of agarose gel: 1%; voltage: 150 V; electrophoresis time: 40 min). And nanodrop instrument (Thermo Fisher Scientific, United States) was used to measure the concentration of DNA. The 16S rRNA gene amplification was carried out in the lab of BGI-WuHan (Beijing Genomic Institute-WuHan Huada Gene Institute) as previous described (Zheng et al., 2017). V4 variable regions of the 16S rRNA gene were amplified by PCR using the primers 515F (5′-GTGCCAGCMGCCGCGGTAA-3′) and 806R (5′-GGACTACHVGGGTWTCTAAT-3′; Langille et al., 2014); 50 μL PCR reaction mixtures contained 30 ng DNA templates, 4 μL PCR primer mixture (515F–806R), 25 μL PCR master mix (NEB Phusion High-Fidelity PCR Master Mix), and appropriate volume of double distilled H_2_O as need. PCR was performed using the following cycling profile: initial denaturation of 98°C for 3 min, followed by 30 cycles of denaturation at 98°C (45 s), annealing at 55°C (45 s), and extension at 72°C for 45 s, and a final extension at 72°C for 7 min. The PCR products were purified using the Ampure XP beads (AGENCOURT) to remove the unspecific product. The Agilent 2100 bioanalyzer instrument (Agilent DNA 1000 Reagents) and real-time quantitative PCR (qPCR) were used to estimate the average molecular length and concentration of the final amplicon library, respectively.

### 16S rRNA Sequencing and Data Analysis

After validation of the library, the qualified libraries were sequenced on the Miseq platform with the sequencing strategy PE250 following the manufacturer’s instructions. High quality reads for subsequent bioinformatic analysis were obtained through in-house pipeline (Huada Gene). In brief, in order to collect clean data from raw data, low quality reads whose mean quality fell below 20 over a 25 bp sliding window based on the Phred algorithm, adapter sequences, ambiguous base (N base), and low complexity reads (default: reads with 10 consecutive same base) were removed. The high quality paired end reads were conjunct to tags using the FLASH analysis tool (v1.2.11^1^; Magoč and Salzberg, 2011) and filtered with QIIME (v1.9.1^2^; Caporaso et al., 2010). UCHIME algorithm (Edgar et al., 2011)^3^ was utilized to remove chimera sequences and obtain the effective tags. The reads were picked to form distinct OTUs at 97% of sequence similarity (Edgar, 2013), and then were classified to different levels based on GreenGenes database with PyNAST software (V1.2; DeSantis et al., 2006). Alpha diversity analysis and beta diversity analysis were calculated by QIIME and visualized by R software (V2.15.3). PCoA was applied to examine dissimilarities in community composition and microbiota abundance was constructed based on the unweighted UniFrac distance metric. All diversity measurements were conducted on OTU tables rarefied to 28,061 sequences per sample. Furthermore, LEfSe^4^ (Segata et al., 2011) was performed by non-parametric factorial KW sum-rank test in order to identify potential microbial biomarkers associated with particular interventions. PICRUSt^5^ is a bioinformatics software package designed to predict the functional profiling of microbial communities based on the 16S rDNA sequences (Langille et al., 2014). STAMP^6^ was used for functional profiling (Parks et al., 2014). Heatmap analysis was carried out to investigate correlations between gut microbiota and diabetes related metabolic indexes.

### Whole-Metagenome Shotgun Sequence Analysis

The extracted DNA from fecal samples of the NCG, DMG, and HLJDD group (*n* = 9, average 3 per group) was sequenced on the Hiseq 4000 Sequencer (Hiseq 4000 SBS Kit, Illumina) with the read lengths 150 bp and insert size of the DNA fragments 350 bp according to the manufacturer’s directions by Huada Gene Institute. Adaptor and low quality reads were discarded from the raw reads, and the remaining reads were filtered in order to eliminate rat host DNA based on the rats reference genome (rn5) using the Burrows-Wheeler Aligner (BWA) algorithm with default parameters (Li and Durbin, 2010). All the high quality sequences from each sample were assembled by SOAPdenovo2 (Luo et al., 2012). The high quality reads from each sample were aligned against the gene catalog by SOAP2 (Qin et al., 2012) in order to determine the abundance of genes. Only genes with ≥2 mapped reads were remained in a sample and the abundance of genes was calculated by counting the number of reads and normalizing by gene length (Qin et al., 2014). The protein sequences of the predicted genes within the KEGG database (Kanehisa et al., 2008) with *E* ≤ 1 × 10^-5^ were searched. The genes were annotated using the KEGG homologs with the lowest e-value. Each protein was allocated to KO (KEGG Orthology group) based on the highest scoring hits with at least one HSP > 60 bits (Bäckhed et al., 2015). The abundance of KO was measured by summing the abundance of genes annotated to the same feature.

### Statistical Analysis

All parameters were expressed as mean ± SD in each group. Significant *p*-values that associated with microbial clades identified by LEfSe were corrected for multiple hypotheses testing using the Benjamini–Hochberg FDR method. Significant *p*-values associated with microbial functions were performed by STAMP, using one-way ANOVA followed by Tukey–Kramer *post hoc* test. Benjamini–Hochberg FDR method was used to correct multiple comparisons, with *p*-value of <0.05 considered to indicate significance. Other statistical tests were performed in GraphPad Prism version 5. Pearson’s correlation coefficient was calculated by SPSS statistics 19.0 to verify the correlations between metabolic/inflammatory indexes and bacterial abundance.

### Sequence Accession Numbers

The sequences generated in this study are available through the NCBI Sequence Read Archive (accession number SRP144903).

## Results

### HPLC Analysis

Marker components contents of the HLJDD extract were measured by HPLC method as described in the part of Section “Materials and Methods.” The HPLC chromatogram of the HLJDD extract is shown in **Supplementary Figure S2**. The contents of geniposide, baicalin, and berberine contained in HLJDD extract were determined to be 7.19, 3.36, and 11.36%, respectively.

### Effect of HLJDD on Body Weight and Metabolic Parameters of T2DM Rats

**FIGURE 1:**
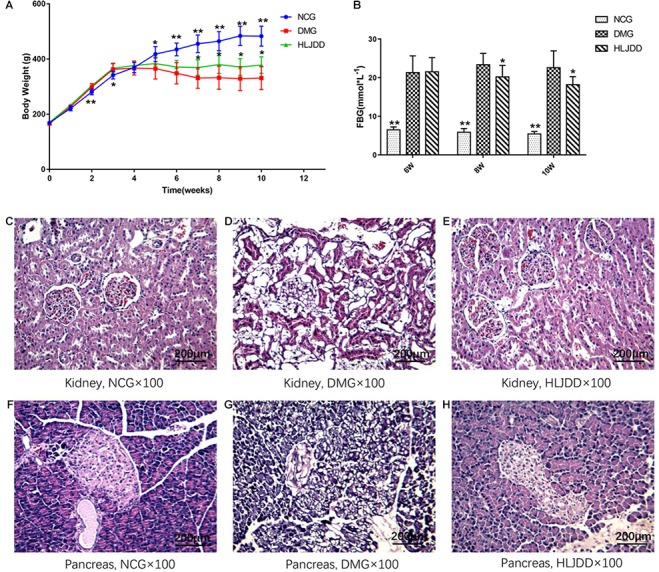
Effects of HLJDD on body weight gain, fasting blood glucose, and the morphology of the kidney and pancreas in rats. **(A)** Body weight gains and **(B)** FBG over the 10-week experiment. **(C–H)** Micrographs of rat kidney and pancreas specimens from NCG, DMG, and HLJDD treatment groups. Values are expressed as means ± SD. Differences were assessed by ANOVA and denoted as follows: ^∗∗^*p* < 0.01, ^∗^*p* < 0.05 vs. DMG (*n* = 8).

**FIGURE 2:**
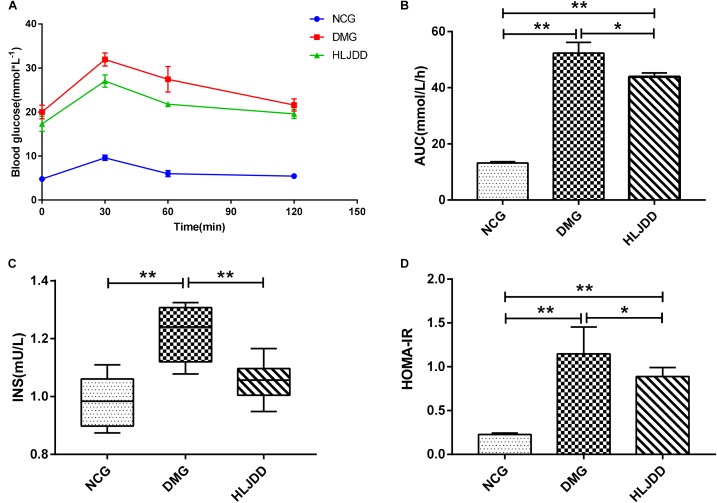
Intervention of HLJDD improves oral glucose tolerance and decreases serum insulin levels. **(A)** Oral glucose tolerance test; **(B)** AUC calculating from the glucose concentrations after glucose load; **(C)** serum insulin levels determined after treatment for 4 weeks; and **(D)** HOMA-IR index calculating from the following formula: HOMA-IR = FINS × FBG/22.5. Values are expressed as means ± SD. Differences were assessed by ANOVA and denoted as follows: ^∗∗^*p* < 0.01, ^∗^*p* < 0.05 vs. DMG (*n* = 8).

To evaluate the effects of HLJDD on T2DM rats, we monitored the body weight and metabolic parameters of rats in all groups. As expected, rats in DMG and HLJDD groups that fed with HFD gained more weight than those in NCG. After intraperitoneal injection of STZ in the fourth week, the body weight of rats in DMG and HLJDD group gradually decrease, and particularly, the weight loss in DMG were more significant than the weight loss in HLJDD group (**Figure 1A**). STZ-induced diabetic rats in DMG showed significant hyperglycemia when compared with NCG (**Figure 1B**), but the FBG of rats in HLJDD group was significantly decreased after 2 weeks’ and 4 weeks’ intervention of HLJDD (the 8th week and 10th week, respectively, in **Figure 1B**). Besides, we also explored the levels of lipids (TG, TC, LDL-C, HDL-C), some inflammatory factors (IL-1β, IL-6, CRP, MDA), some antioxidative enzymes (SOD, GSH-Px), and liver function indexes (ALT, AST, TBA, TBIL, DBIL), which can help explaining the influences of HLJDD on T2DM. Compared with NCG, DMG had notably higher serum concentrations of TG, LDL-C, ALT, TBA, TBIL, DBIL, IL-1β, IL-6, MDA, and CRP, while concentrations of HDL-C, SOD, and GSH-Px were lower in rats of DMG than NCG (**Table 1**). HLJDD intervention remarkedly helped to correct lipid metabolism disorders, alleviate systematic inflammation and improve anti-oxidant ability. Moreover, the organ indexes (**Supplementary Table S2**) showed that indexes of liver, kidney, and pancreas in DMG rats increased significantly when compared with rats in NCG. The pathological sections (**Figures 1C–H**) indicated that both the renal tubule epithelium and pancreas in DMG had obvious fatty degeneration and fat vacuoles. HLJDD intervention can decrease the compensatory insulin secretion in DMG rats (**Figure 2C**), reduce the fat vacuoles and compensatory hypertrophy of kidney as well as pancreas. Additionally, the responses of plasma glucose to OGTT are shown in **Figure 2A**, indicating that rats in DMG have higher glucose levels after glucose administration when compared with NCG. The AUC of plasma glucose calculated from the OGTT (**Figure 2B**) was significantly decreased in HLJDD rats when compared with DMG. Furthermore, levels of FINS and HOMA-IR in three groups (**Figures 2C,D**) also revealed that HLJDD improved impaired glucose tolerance in STZ-induced T2DM rats.

**Table 1:** Comparison of metabolic index levels in rats among the different groups.

Group	NCG	DMG	HLJDD
TG (mmol/L)	0.78 ± 0.21**	2.88 ± 1.71	1.18 ± 1.03*
TC (mmol/L)	1.66 ± 0.29	2.40 ± 1.00	1.51 ± 0.62*
LDL-C (mmol/L)	0.13 ± 0.05**	0.56 ± 0.38	0.22 ± 0.20*
HDL-C (mmol/L)	0.67 ± 0.10	0.58 ± 0.38	0.62 ± 0.13
AST (U/L)	105.00 ± 17.89	99.11 ± 40.92	91.00 ± 17.13
ALT (U/L)	38.00 ± 16.46**	66.22 ± 10.79	59.78 ± 6.69
TBA (μmol/L)	7.21 ± 3.35**	56.92 ± 25.54	19.62 ± 12.06*
TBIL (μmol/L)	1.57 ± 0.33**	2.26 ± 0.56	2.17 ± 0.30
DBIL (μmol/L)	0.52 ± 0.26**	1.87 ± 0.40	1.58 ± 0.39
IL-1β (pg/mL)	32.21 ± 13.44*	50.73 ± 33.52	36.38 ± 8.37**
IL-6 (pg/mL)	116.03 ± 14.76*	127.64 ± 8.80	123.70 ± 13.41
MDA (U/mL)	8.07 ± 1.51*	10.33 ± 1.89	7.52 ± 1.06**
SOD (U/mL)	148.20 ± 12.07**	130.57 ± 9.38	137.76 ± 4.78*
GSH-Px (U/mL)	2184.26 ± 465.93	1972.13 ± 424.51	2671.51 ± 645.17*
CRP (mg/L)	77.92 ± 0.52*	79.11 ± 1.30	77.80 ± 0.89*

*Results were expressed as mean ± SD, n = 8. ^∗∗^p < 0.01, ^∗^p < 0.05 vs. DMG.*

### Overall Structure Modulation of Gut Microbiota Among Different Groups

**FIGURE 3:**
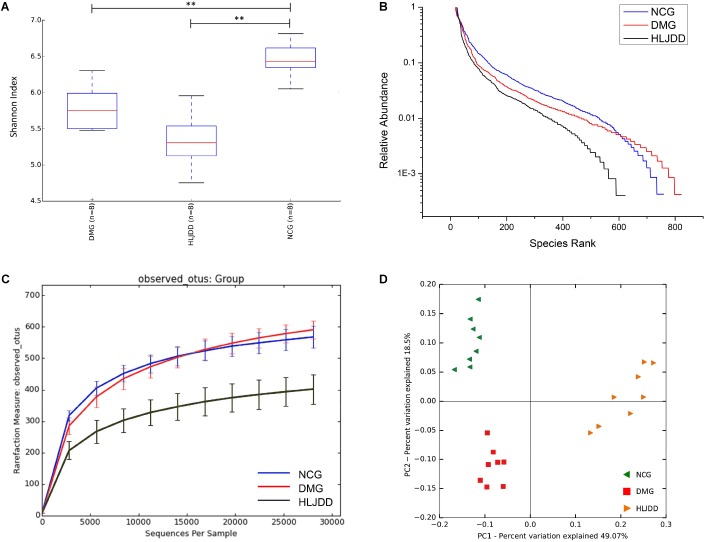
Diversity of fecal microbiota among the NCG, DMG, and HLJDD group. **(A)** Shannon index (data expressed as mean ± SD); **(B)** rank abundance curve; **(C)** rarefaction analyses; and **(D)** unweight UniFrac-based PCoA. Differences were assessed by ANOVA followed by Tukey’s *post hoc* test and denoted as follows: ^∗∗^*p* < 0.01 (*n* = 8).

The V4 region of bacterial 16S rRNA was analyzed by MiSeq platform to examine the impact of HLJDD on gut microbiota composition. After removing unqualified sequences, a total of 986,135 raw reads and an average of 41,088 ± 621 reads per sample were obtained, and the length was 250 bp. Following chimera checking, a total of 802,410 effective reads, with an average of 33,434 ± 1099 per sample, remained for downstream analysis. Common microbial α-diversity indexes, including Shannon index, rank-abundance curve, and rarefaction curve, were evaluated (**Figures 3A–C**). As shown in **Figure 3A**, Shannon indexes of the DMG and HLJDD were significantly lower than NCG (*p* < 0.01). And the diversity of HLJDD was also the lowest one in rank-abundance curve (**Figure 3B**). Moreover, the quantity of observed OTUs in the HLJDD group was lower than the other two groups (**Figure 3C**). The phenomenon that rarefaction curve analysis already reached stable values in the current sequencing, demonstrated that the sequencing depth had covered rare new phenotypes and species as much as possible. In conclusion, DMG displayed a decreasing biodiversity when compared with NCG and HLJDD group was the lowest one.

**FIGURE 4:**
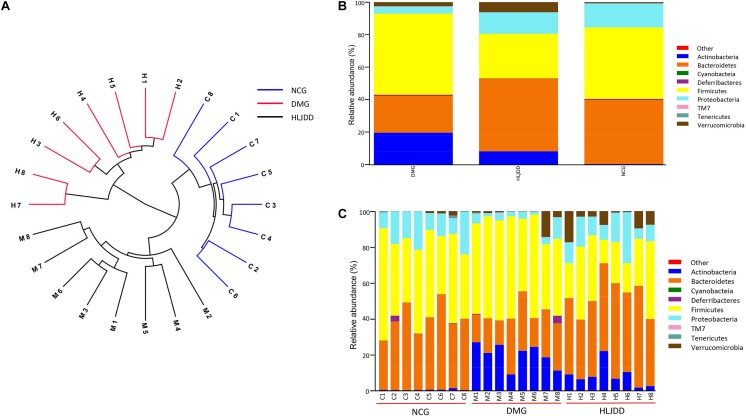
Comparison of the feces microbial community among three groups. **(A)** Multivariate analysis of variance from unweight UniFrac-based PCoA matrix scores. **(B)** Bacterial taxonomic profiling in the phylum level of gut microbiota at individual level. **(C)** Bacterial taxonomic profiling in the phylum level of gut microbiota based on different groups.

UniFrac-based PCoA of microbiota composition showed a noticeable clustering for each group (**Figure 3D**). Multivariate ANOVA of unweighted UniFrac metrics displayed an obvious separation among the three groups (**Figure 4A**). Bacterial sequences from the three groups were analyzed at the phylum level, and the top 10 phyla in the relative abundance of gut microbiota in different groups and each sample were showed as follows (**Figures 4B,C**). The relative abundance of four primary phyla (Bacteroides, Firmicutes, Proteobacteria, and Actinobacteria) in each group is listed in **Table 2**. The relative abundance of Bacteroidetes and Proteobacteria significantly decreased in DMG compared with NCG, and both phyla of microbiota increased after treated with HLJDD compared to DMG. Besides, B/F in NCG, DMG, and HLJDD were 0.89, 0.45, and 1.63, respectively. Taxonomic profiling demonstrated that HLJDD treatment could modulate the gut composition of rats with T2DM into a similar level of NCG.

**Table 2:** Relative abundance of four major bacteria phyla among three groups.

Major phyla	Relative abundance (%)			*p*-value		
	NCG	DMG	HLJDD	DMG vs. NCG	DMG vs. HLJDD	NCG vs. HLJDD
Bacteroidetes	39.31 ± 8.49	22.59 ± 7.53	44.71 ± 7.82	0.001	<0.001	0.381
Firmicutes	43.99 ± 9.93	49.76 ± 8.52	27.45 ± 11.53	0.496	<0.001	0.009
Proteobacteria	14.96 ± 5.95	4.34 ± 3.32	13.33 ± 7.16	0.004	0.013	0.883
Actinobacteria	0.30 ± 0.31	19.75 ± 6.63	8.18 ± 6.30	<0.001	<0.001	0.019

*p-values were calculated using Tukey’s honest significant difference test. p-values < 0.05 were considered statistically significant.*

### Key Phenotypes Responding to the HLJDD Treatment in T2DM

**FIGURE 5:**
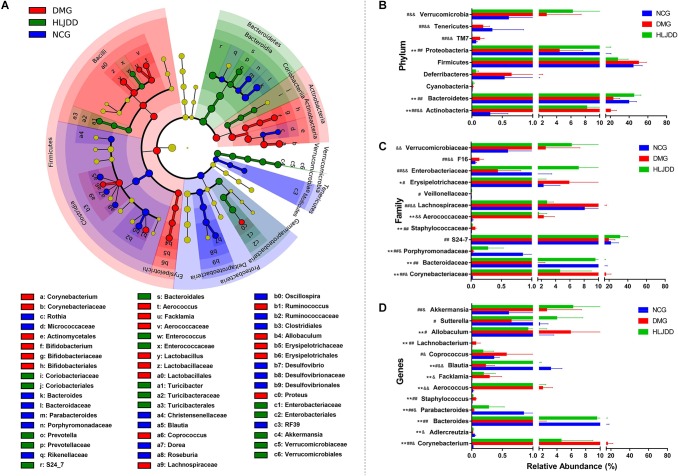
Taxonomic differences of fecal microbiota between NCG, DMG, and HLJDD group. **(A)** Taxonomic cladogram obtained by LEfSe. Differences are represented by the color of the most abundant class. (Red indicating DMG, green HLJDD, and blue NCG.) The diameter of each circle is proportional to the taxon’s abundance. Comparison of relative abundance at the phylum **(B)**, family **(C)**, and genus **(D)** levels among three groups. Significant values between groups were calculated by Mann–Whitney *U*-test: NCG vs. DMG (^∗^*p* < 0.05, ^∗∗^*p* < 0.01); DMG vs. HLJDD (^#^*p* < 0.05, ^##^*p* < 0.01); NCG vs. HLJDD (^&^*p* < 0.05, ^&&^*p* < 0.01) (*n* = 8).

To further investigate differences in the microbiome among NCG, DMG, and HLJDD group, we used LEfSe to identify the specific altered bacterial phenotypes (from phylum to genus). The cladogram showed the dominant bacteria in each group (**Figure 5A**). A total of 69 bacteria changed significantly among the three groups, with an LDA score log 10 > 3 (**Supplementary Figure S3**). Constitutions of gut bacterial species among the three groups showed apparent variation. At the general level, differential microbial lineages of the NCG included the *Desulfovibrio*, *Blautia*, *Roseburia*, *Bacteriodes*, and *Parabacteriodes*. Clades associating with DMG included the *Facklamia*, *Aerococcus*, *Corynebacterium*, *Bifidobacterium*, and *Allobaculum*. Moreover, the following microbiota was the most abundant in the HLJDD group: the *Prevotella* and *Akkemansia*.

The changes in the microbiota after HLJDD treatment were also exhibited at different taxon levels including phylum, family and genus (**Figures 5B–D**). In summary, compared with NCG, DMG showed a decreased level of SCFAs-producing bacteria, such as *Adlercreutzia*, Porphyromonadaceae (including *Parabacteroides*), and Lachnospiraceae (including *Blautia*; **Figures 5C,D**). At the same time, opportunistic pathogens like Corynebacteriaceae (including *Corynebacterium*), Staphylococcaceae (including *Staphylococcus*), and Aerococcaceae (including *Aerococcus* and *Facklamia*) were enriched in DMG (**Figures 5C,D**). These changes in microbiome indicated the gut microbiota dysbiosis in T2DM. Collectively, compared with DMG, there is an increased level of SCFAs-producing bacteria, such as *Adlercreutzia*, Porphyromonadaceae (including *Parabacteroides*), Lachnospiraceae (including *Blautia*; **Figures 5C,D**), and the opportunistic pathogens mentioned above decreased in HLJDD group. What’s more, the Bacterioidetes (including *Akkemansia*) enriched in the HLJDD group compared with both NCG and DMG.

### Correlation of Gut Microbiota With Metabolic Parameters of T2DM

**FIGURE 6:**
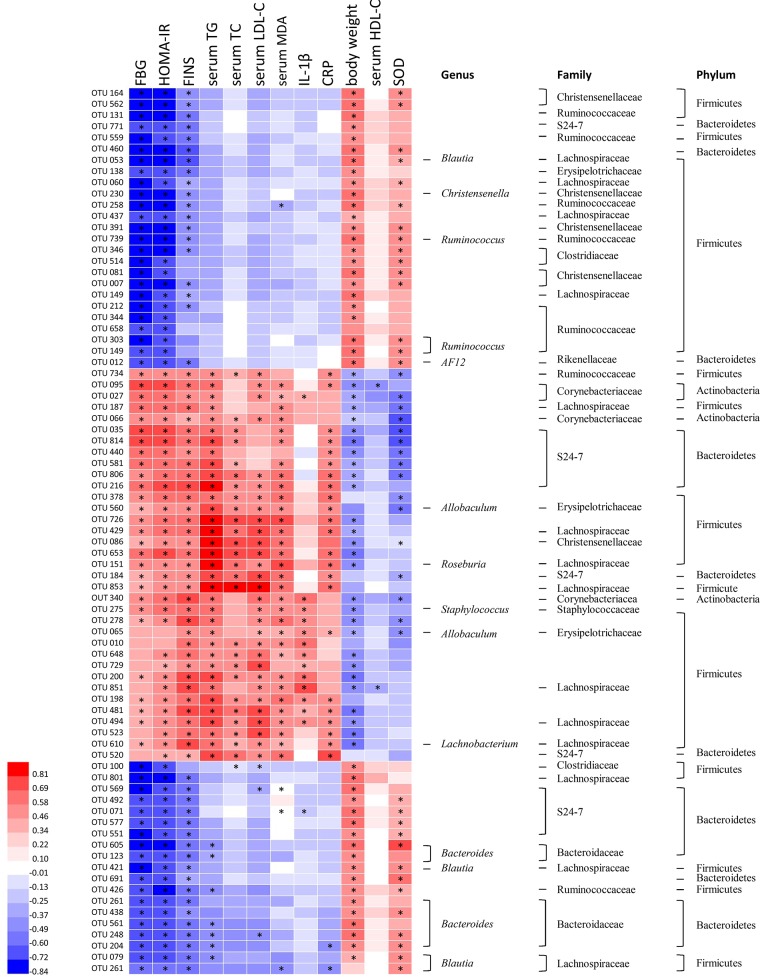
Correlation analysis between gut microbiota and T2DM-related biomedical factors in rats. Heatmap of correlation between the alterations in gut microbial population and the changes in host parameters related to blood glucose, lipid peroxidation, inflammatory factor, and antioxidant enzyme activities. Pearson correlation values were used for the matrix. Significant correlations were noted by adjusted *^∗^p* < 0.05.

Correlation heatmap analysis was applied to assess the association between gut microbiota and T2DM related indexes (body weight, FBG, HOMA-IR, FINS, TG, TC, HDL-C, MDA, CRP, SOD, IL-1β; **Figure 6**). Based on this heatmap, we found that *Blautia*, *Christensenella*, *Ruminococcus*, *Bacteroides*, and *AF12* were negatively correlated with metabolic indexes (except body weight, HDL-C and SOD), while other bacteria (including *Allobaculum*, *Roseburia*, *Staphylococcus*, and *Lachnobacterium*) were positively associated with metabolic indexes (except body weight, HDL-C, and SOD).

### Microbial Metabolic Functions Associated With HLJDD Treatment in T2DM Rats

**FIGURE 7:**
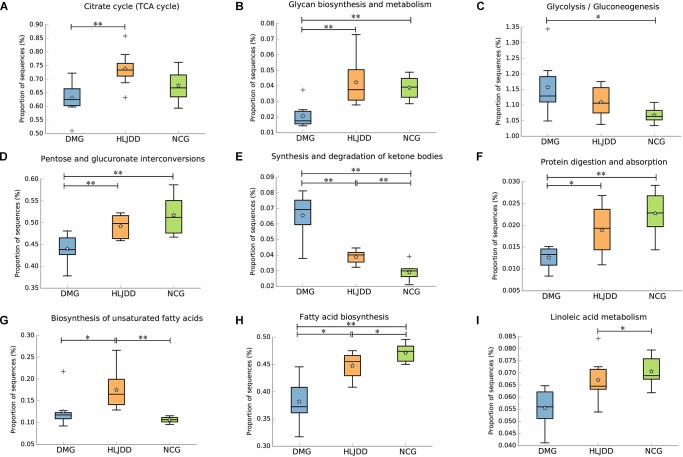
Inferred gut microbiome functions by PICRUSt from 16S rRNA gene sequences among the three groups (NCG, DMG, HLJDD). **(A)** Citrate cycle. **(B–F)** Metabolism of carbohydrates. **(G–I)** Lipid metabolism. Box plots denote the top quartile, median, and bottom quartile, and white stars mean the average value as well as the “+” means the outlier. All the differences were analyzed using one-way ANOVA followed by Tukey–Kramer *post hoc* test; Benjamini–Hochberg FDR method was used to correct multiple comparisons (^∗^*p* < 0.05, *^∗∗^p* < 0.01).

**FIGURE 8:**
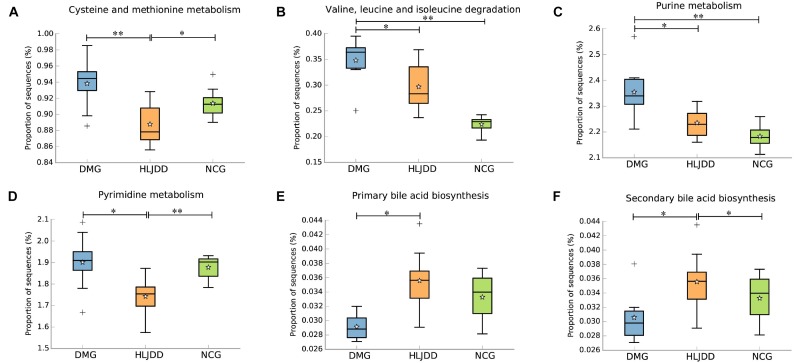
Inferred gut microbiome functions by PICRUSt from 16S rRNA gene sequences among the three groups (NCG, DMG, HLJDD). **(A,B)** The metabolism and biosynthesis of amino acids. **(C,D)** Nucleic acid metabolism. **(E,F)** Bile acid biosynthesis. Box plots denote the top quartile, median, and bottom quartile, and white stars mean the average value as well as the “+” means the outlier. All the differences were analyzed using one-way ANOVA followed by Tukey–Kramer *post hoc* test; Benjamini–Hochberg FDR method was used to correct multiple comparisons (^∗^*p* < 0.05, *^∗∗^p* < 0.01).

PICRUSt analysis based on 16S rRNA gene sequence data was used to investigate the gut microbiome functions related to HLJDD treatment. We also adopted STAMP to identify relevant microbial functions associated with T2DM and HLJDD treatment. PCA analysis based on the PICRUSt analysis (KEGG level) was also generated. PCA plots showed an obvious clustering of microbiota composition among three groups (**Supplementary Figure S4**). Decreased basic metabolism was presented in DMG compared with the NCG and HLJDD group (**Figures 7A,B,F**), such as TCA cycle, glycan biosynthesis, and metabolism. As regards the glucose metabolism, there was a significant rise in DMG in pathways like synthesis and degradation of ketone bodies, glycolysis/gluconeogenesis, and a decrease in pentose and glucuronate interconversions in DMG compared to the other two groups (**Figures 7C–E**). The current situation of basic metabolism and glucose metabolism suggested that the gut microbiota in DMG was in a mess and the disorder was improved after HLJDD treatment. In addition, there were differences in lipid metabolism (biosynthesis of unsaturated fatty acids, fatty acid biosynthesis and linoleic acid metabolism) among the three groups (**Figures 7G–I**). A portion of amino metabolism and nucleotide metabolism was significantly upregulated in DMG compared with NCG and HLJDD (**Figures 8A–F**). Other metabolisms, such as bile acid biosynthesis, were enriched in HLJDD group compared with DMG. In summary, the results demonstrated that T2DM led to a gut microbiota disorder and HLJDD treatment could reverse the gut microbiome functions to a similar level of NCG.

**FIGURE 9:**
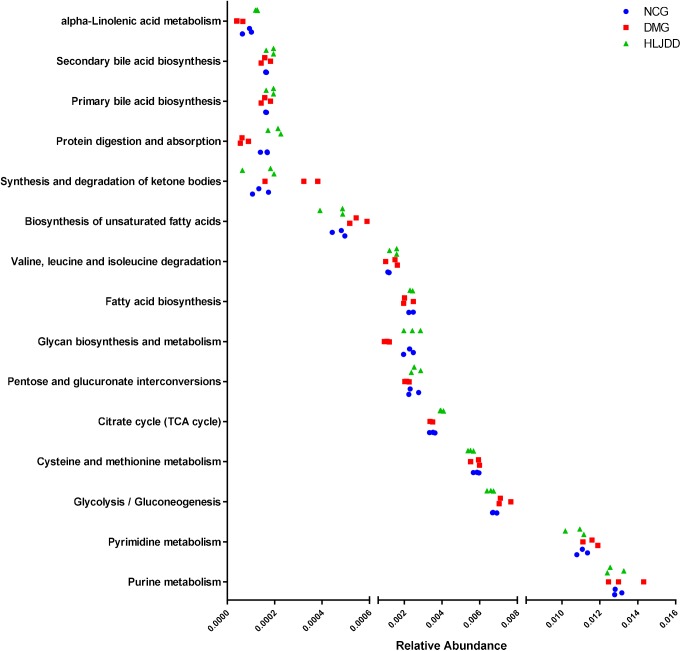
Shotgun sequencing validates the predicted microbial metabolic trends in a subset of fecal samples from the three group. Among the 15 microbial metabolic pathways, 14 retained the same under- or over-abundance trend as that were predicted from 16S gene sequencing, except the valine, leucine, and isoleucine degradation.

The inferred functions were validated by shotgun metagenomic sequencing based on a subset of nine fecal samples from the three groups (**Figure 9**). A total of 347,310,358 qualified shotgun sequences were obtained with an average of 38,590,040 ± 321,403 reads. As shown in **Figure 9**, 14 metabolic pathways retained the same under- or over-abundance trend as that were predicted from 16S gene sequencing, except the valine, leucine, and isoleucine degradation.

## Discussion

The gut microbiota of humans and rats has a high similarity although there are some important differences (Krych et al., 2013). Under the circumstances, the preclinical model of T2DM is a good alternative to investigate the correlation between the intestinal microbiota and T2DM (Sah et al., 2016). More than 40 experimental animal models of T2DM have been established (Sah et al., 2016). The STZ and diet-induced T2DM model, which shared most features with human T2DM (King and Bowe, 2016), which offers an inexpensive and reproducible model that used in exploring the relationships of T2DM, HLJDD, and gut microbiome. Thus, in this study, a single intraperitoneal injection of STZ was performed after 4 weeks of HFD feeding to induce T2DM in rats. The FBG, FINS, HOMA-IR, and pathological results of DMG demonstrated that the rats in DMG developed similar symptoms to human T2DM. At the same time, the results of HLJDD group showed that HLJDD treatment could lower blood glucose, which is consistent with previous studies (Jin et al., 2010; Zhang et al., 2014). In order to illustrate the underlying hypoglycemic mechanisms of HLJDD, we speculated that HLJDD ameliorated T2DM via the interaction with gut microbiota. Hence, the 16S rRNA gene sequencing and shotgun metagenomic sequencing were conducted. In the current study, rarefaction curve analysis has already reached a stable value, indicating that the sequencing has satisfied the most diversities and covered rare new phenotypes. Meanwhile, a decrease of biodiversity was observed in DMG compared with NCG. We found the total abundance of gut microbiota was further decreased after HLJDD administration, which could be due to the antibacterial effect of berberine, an effective component of HLJDD (Chu et al., 2014).

Bacteroidetes and Firmicutes are two main phyla in the gut microbiota in rats as well as humans. It is reported that propionic acid is the main fermentation product of Bacteroidetes, which can significantly inhibit cholesterol and fatty acid synthesis in the liver of mice (Wright et al., 1990). Additionally, the B/F ratio increased when resveratrol improved the gut microbiota dysbiosis induced by the HFD in a previous study (Qiao et al., 2014). In our study, HLJDD treatment also reversed the decrease of B/F ratio in DMG. And then, the effects of HLJDD acting on the microbiome were assessed by comparing specific bacteria among three groups. Our results showed that gut microbiota of DMG was mainly associated with decreased levels of SCFAs-producing bacteria, including *Adlercreutzia*, *Parabacteroides*, and *Blautia*, and these bacteria were enriched after HLJDD treatment. SCFAs, including acetate, propionate, and butyrate, are the primary end products from the degradation of carbohydrates by gut microbiota in the intestine (Hold, 2014). SCFAs could regulate lipid and carbohydrate metabolism in the liver, lower blood glucose, and lipid levels (Tremaroli and Bäckhed, 2012), improve glucose homeostasis and insulin sensitivity by beneficially modulating the function of skeletal muscle, liver, and adipose tissue (Canfora et al., 2015). It is reported that the abundance of *Adlercreutzia* and *Parabacteroides* is negatively correlated with IBD (Shaw et al., 2016), obesity (Henning et al., 2017), and T2DM (Wei et al., 2018). *Blautia*, belonging to the family Lachnospiraceae of phylum Firmicutes, could ferment carbohydrates and produce acetate and butyrate, and then produce energy for organism (Park et al., 2013). Many studies illustrated that *Blautia* was negatively correlated with age, obesity and T2DM (Kasai et al., 2015; Rondanelli, 2015; Inoue et al., 2017). Henning et al. (2017) found that decaffeinated green and black tea polyphenols reduced weight gain and enriched *Blautia* in diet-induced obese mice.Yang et al. (2015) demonstrated that xylooligosaccharide supplementation could alter gut bacteria, enrich *Blautia*, and improve glucose control in prediabetic adults. It is noteworthy that although there was no difference in level of *Akkermansia* between NCG and DMG, it increased significantly after HLJDD treatment, which may be related to the hypoglycemic and anti-inflammatory effect of HLJDD. *Akkermansia*, which belongs to Verrucomicrobiaceae family in Verrucomicrobia phylum, was reported to be negatively related to obesity, metabolic syndrome, and T2DM (Schneeberger et al., 2015). As a mucus-degrading bacteria located in the mucous layer, *Akkermansia* could increase the secretion of choline in the intestine, regulate inflammation, intestinal barrier, and secretion of peptides (Everard et al., 2013). In addition, it is reported that metformin and dietary polyphenols could promote the growth of *Akkermansia* in HFD-induced obesity. Furthermore, conditioned pathogens including *Facklamia*, *Aerococcus*, *Staphylococcus*, and *Corynebacterium* were enriched in rats in DMG and modified after the oral administration of HLJDD, indicating that the relative abundance of these bacteria might be associated with the pathological status of T2DM (Koch et al., 2014; Wolcott et al., 2016; Rahmati et al., 2017). The conditioned pathogens mentioned above, which were positively correlated with inflammation in body, might be the targeted bacteria involved the progress that HLJDD alleviated T2DM (Lenherr et al., 2014). At the same time, *Allobaculum*, *Lachnobacterium*, and *Coprococcus*, which enriched in DMG compared to NCG, were reversed by HLJDD treatment. And the change tendency of *Lachnobacterium* and *Coprococcus* was consistent with the previous study (Wei et al., 2018). However, *Allobaculum*, one of the SCFAs-producing bacteria, was reported to increase in HFD-induced obesity rats treated with berberine and metformin (Zhang et al., 2015). Besides, numerous studies also illustrated that *Allobaculum* increased in some diseases like hypercholesteremia and obesity (Lee et al., 2015; Liu et al., 2016). The trend of *Allobaculum* in health remains controversial and needs further investigation. Taking the above into consideration, our results showed a decrease of SCFAs-producing bacteria and an increased of conditioned pathogens in DMG, which could be modified to a composition similar to NCG by HLJDD treatment.

The functional capabilities of the microbial communities were analyzed by PICRUSt. A wide range of biological functions were influenced by T2DM and HLJDD treatment and the inferred metagenomes from the 16S data were validated by shotgun metagenomic sequencing. Our study showed an increase in glycolysis/gluconeogenesis, amino metabolism, and nucleotide metabolism in T2DM and the above functions were modified after HLJDD treatment. Previous study also showed that there was an increase in glycolysis/gluconeogenesis and amino metabolism in T2DM (Inoue et al., 2017). The degradation of cholesterol is one of the most important pathways of bile acid synthesis. Recent studies showed that bile acids synthesis was associated with modulation of gut microbiota and played a vital role in maintaining glucose, lipid and energy homeostasis (David et al., 2014). In our study, the decrease of bile acid biosynthesis in DMG might be related to glucose and lipid metabolism disorder. There was an increased tendency of bile acid biosynthesis in HLJDD group compared with DMG, which was in accordance with physiological indexes such as levels of TG, TC, LDL, and FBG. As for amino metabolism, it was reported that high levels of homocysteine (an important intermediate metabolite produced during cysteine and methionine metabolism) in the blood have been linked to increasing risk of T2DM, diabetic nephropathy, premature coronary heart disease, and stroke (Huang et al., 2013; Borghi, 2015). Purine and pyrimidine are crucial substrate for ribonucleic acids derivation (i.e., DNA and RNA) and also necessary in promoting the cellular metabolism by producing nucleotides (i.e., ATP, ADP, and AMP; Rengaraj et al., 2013). Uric acid is an important metabolic end product of purine. Recent reports showed that serum uric acid played an important role in the occurrence development and prognosis of cardiovascular diseases and T2DM (Kanbay et al., 2013). Additionally, the high concentration of pyrimidine and cytidine can cause endothelial cell damage in renal vessels, resulting in abnormal renal hemodynamics and leading to diabetes and diabetic nephropathy (Takezako et al., 2001). Collectively, the functional variation of microbiota among the NCG, DMG, and HLJDD group indicated that HLJDD treatment could regulate the disordered metabolic pathways in T2DM rats, repairing the abnormal microbiota function to a normal state.

## Conclusion

In summary, our study aimed at discovering the changes of the gut microbiome composition and function in animal T2DM model with and without HLJDD treatment. It was indicated that HLJDD could improve the hyperglycemia, the metabolic disturbance of lipids, and inflammation, shape the microbiome, and restore the dysregulated microbiota function in T2DM rats into a normal condition in T2DM rats. More specifically, HLJDD treatment increased the abundance of SCFAs-producing and anti-inflammatory bacteria such as *Parabacteroides*, *Blautia*, and *Akkermansia*, and decreased the amount of conditioned pathogenic bacteria (e.g., *Aerococcus*, *Staphylococcus*, and *Corynebacterium*) at the same time. Based on the mentioned above, it was suggested that regulating the gut microbiota using TCM may become a potential therapeutic strategy of T2DM and the modification of gut microbiota might be one of the proposed mechanisms of HLJDD treatment to improve diabetes. Our findings provided a novel insight into the role that HLJDD exerted systemically hypoglycemic activities from the perspective of gut microbiota. Additionally, the results need more verifications which can gain by exploring the changes of gut microbiota in human with HLJDD treatment.

## Author Contributions

QL and ZX conceived the original idea, and designed and supervised the experiments, analysis, and writing. MC and ZL carried out the sample collection and data analysis and drafted the manuscript. BL and MW revised the final draft of the paper. LL, SZ, and DL performed the technical procedures and participated in the animal surgeries and the sample collection. All authors read and approved the final manuscript.

## Conflict of Interest Statement

The authors declare that the research was conducted in the absence of any commercial or financial relationships that could be construed as a potential conflict of interest.

## Footnotes

1 http://ccb.jhu.edu/software/FLASH/

2 http://qiime.org/scripts/split_libraries_fastq.html

3 http://www.drive5.com/usearch/manual/uchime_algo.html

4 http://huttenhower.sph.harvard.edu/lefse/

5 http://picrust.github.io/picrust/

6 http://kiwi.cs.dal.ca/Software/STAMP

## Abbreviations

ALT: alanine aminotransferase
ANOVA: analysis of variance
AST: aspartate aminotransferase
AUC: the area under the curves
B/F: ratios of Bacteroidetes to Firmicutes
CMC-Na: sodium carboxymethyl cellulose
CRP: C-reactive protein
DBIL: direct bilirubin
DMG: type 2 diabetic model group
ELISA: enzyme-linked immune sorbent assay
FBG: fasting blood glucose
FDR: false discovery rate
FINS: fasting insulin
FLASH: fast length adjustment of short reads
GSH-Px: glutathione peroxidase
HDL-C: high-density lipoprotein cholesterol
HFD: high-fat diet
HLJDD: Huang-Lian-Jie-Du-Decoction
HOMA-IR: homeostatic model index-insulin resistant
HPLC: high-performance liquid chromatographic
IACUC: the Institutional Animal Care and Use Committee
IBD: inflammatory bowel disease
IL-1β: interleukin-1 beta
IL-6: interleukin-6
KEGG: the Kyoto Encyclopedia of Genes and Genomes
KO: the Kyoto Encyclopedia of Genes and Genomes Orthology group
KW: Kruskal–Wallis
LDA: linear discriminant analysis
LDL-C: low-density lipoprotein cholesterol
LEfSe: linear discrimination analysis effect size
LPS: lipopolysaccharide
MDA: malonaldehyde
NCBI: National Center of Biotechnology Information
NCG: the control diet group
OGTT: oral glucose tolerance test
OTUs: operational taxonomic units
PCoA: principal coordinates analysis
PCR: polymerase chain reaction
PICRUSt: Phylogenetic Investigation of Communities by Reconstruction of Unobserved States
PyNAST: Python Nearest Alignment Space Termination
QIIME: quantitative insight into microbial ecology
qPCR: real-time quantitative polymerase chain reaction
SCFAs: short chain fatty acids
SD: standard deviation
SOAPdenovo2: Short Oligonucleotide Analysis Package
SOD: superoxide dismutase
SPSS: Statistical Product and Service Solutions
STZ: streptozotocin
T2DM: type 2 diabetes mellitus
TBA: total bile acid
TBIL: total bilirubin
TC: total serum cholesterol
TCA: citrate cycle
TCM: traditional Chinese medicine
TG: triglycerides
