# Supplementary material for: Huang-Lian-Jie-Du-Decoction Ameliorates Hyperglycemia and Insulin Resistant in Association With Gut Microbiota Modulation
Source: Front Microbiol. 2018 Oct 8;9:2380. doi: 10.3389/fmicb.2018.02380 (PMC6186778; doi:10.3389/fmicb.2018.02380)
Supplement: Supplementary file 1 [file Data_Sheet_1.DOCX]

Supplementary Material

Huang-Lian-Jie-Du-Decoction ameliorates hyperglycemia and insulin resistant in association with gut microbiota modulation

Mingyi Chen, Ziqiong Liao, Biyu Lu, Mengxia Wang, Lei Lin, Shaobao Zhang, Yuan Li, Deliang Liu, Qiongfeng Liao^*^ and Zhiyong Xie^*^

*** Correspondence:**
Zhiyong Xie
[xiezy2074@yahoo.com](mailto:xiezy2074@yahoo.com)
Qiongfeng Liao
[liaoqf2075@yahoo.com](mailto:liaoqf2075@yahoo.com)

# Supplementary Figures and Tables

## Supplementary Figures
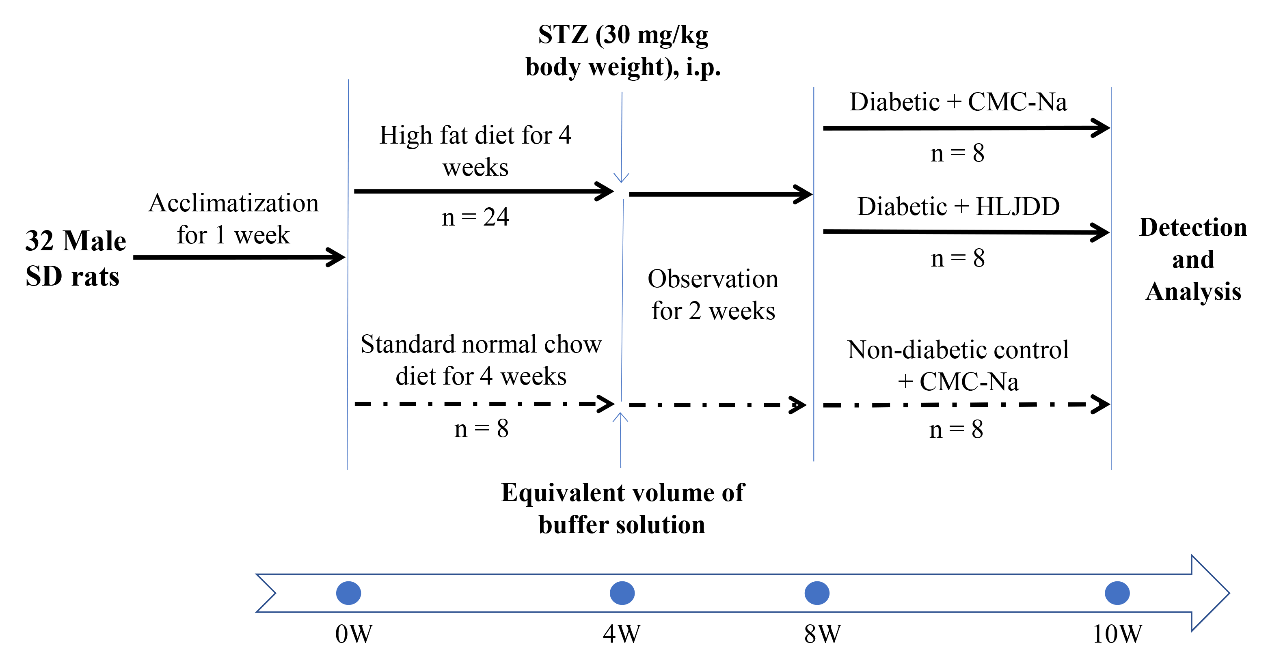


**Supplementary Figure 1.** The flow chart of the experiment.


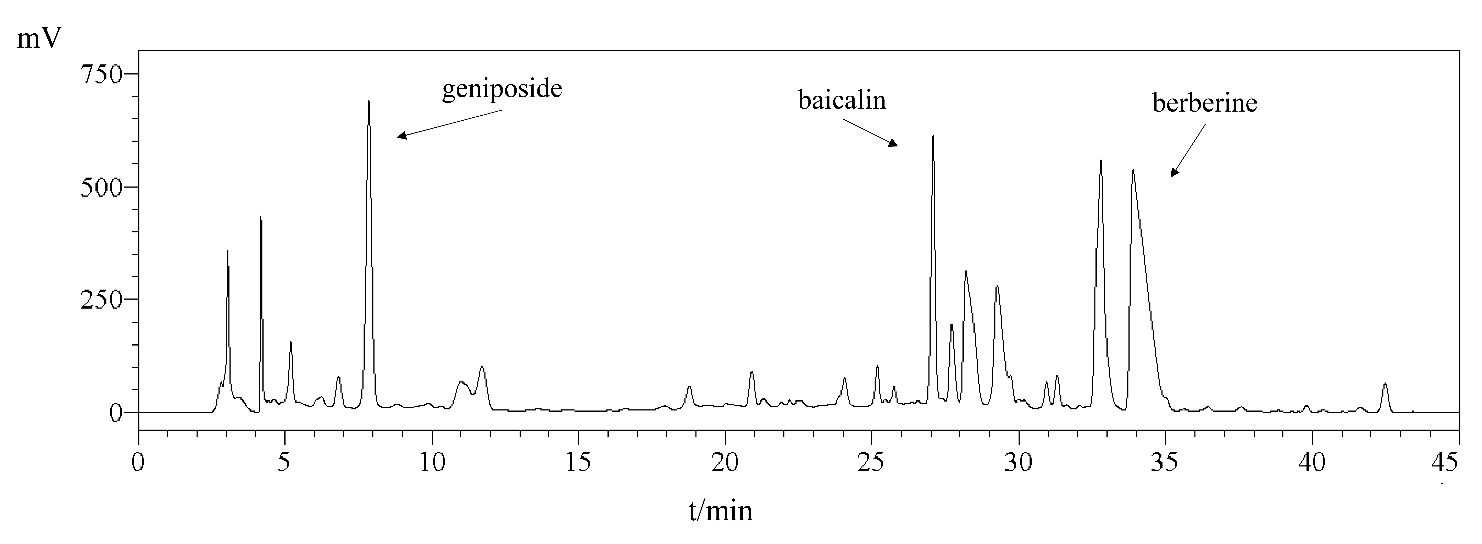


**Supplementary Figure 2.** HPLC chromatogram of the Huang-Lian-Jie-Du-Decoction extract.


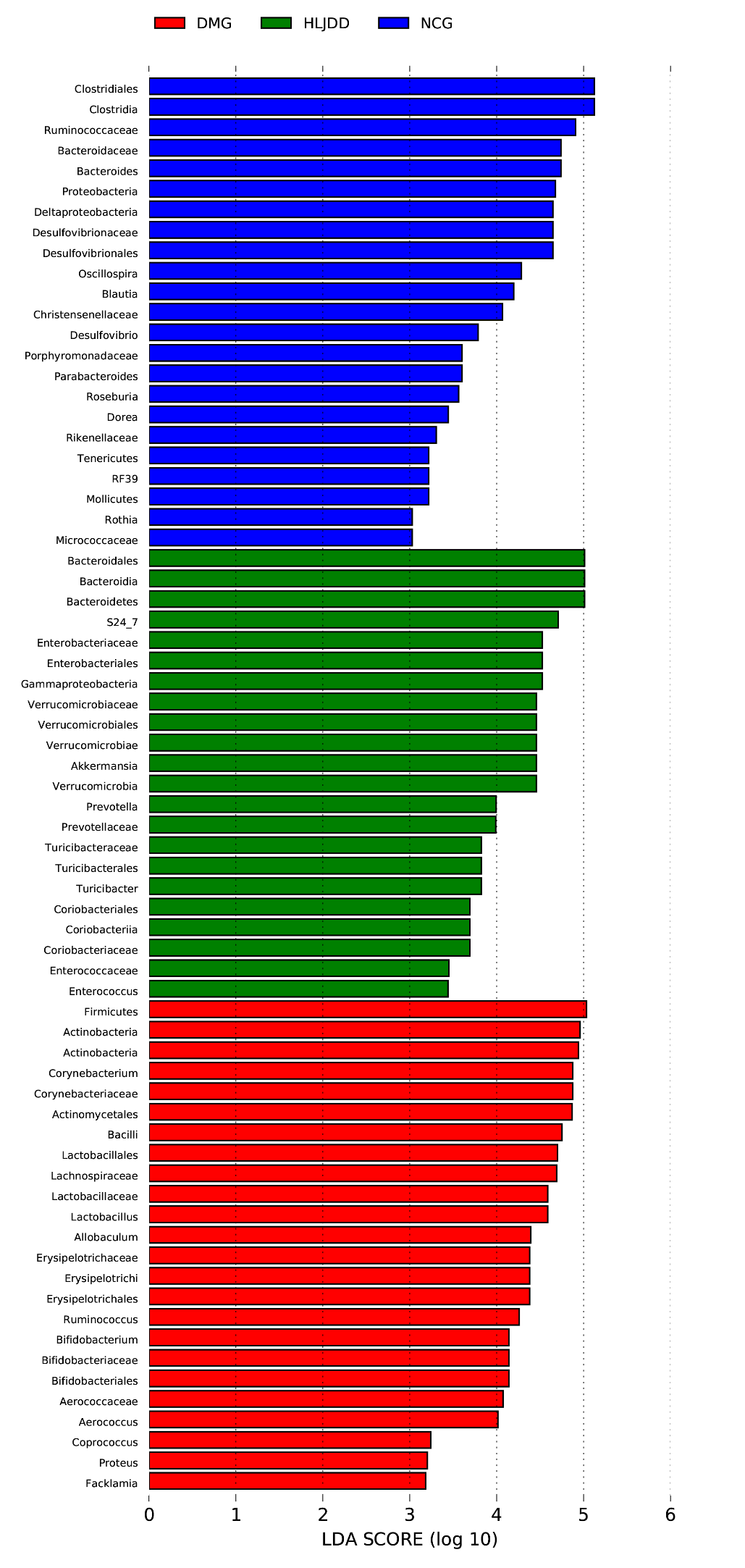


**Supplementary Figure 3.** LEfSe rank plots of differentially abundant microbial clades in gut microbiome associated with NCG, DMG and HLJDD group. LDA scores for differentially abundant microbial clades in stool among three groups and the threshold on the logarithmic LDA score for discriminative feature is > 3.0.


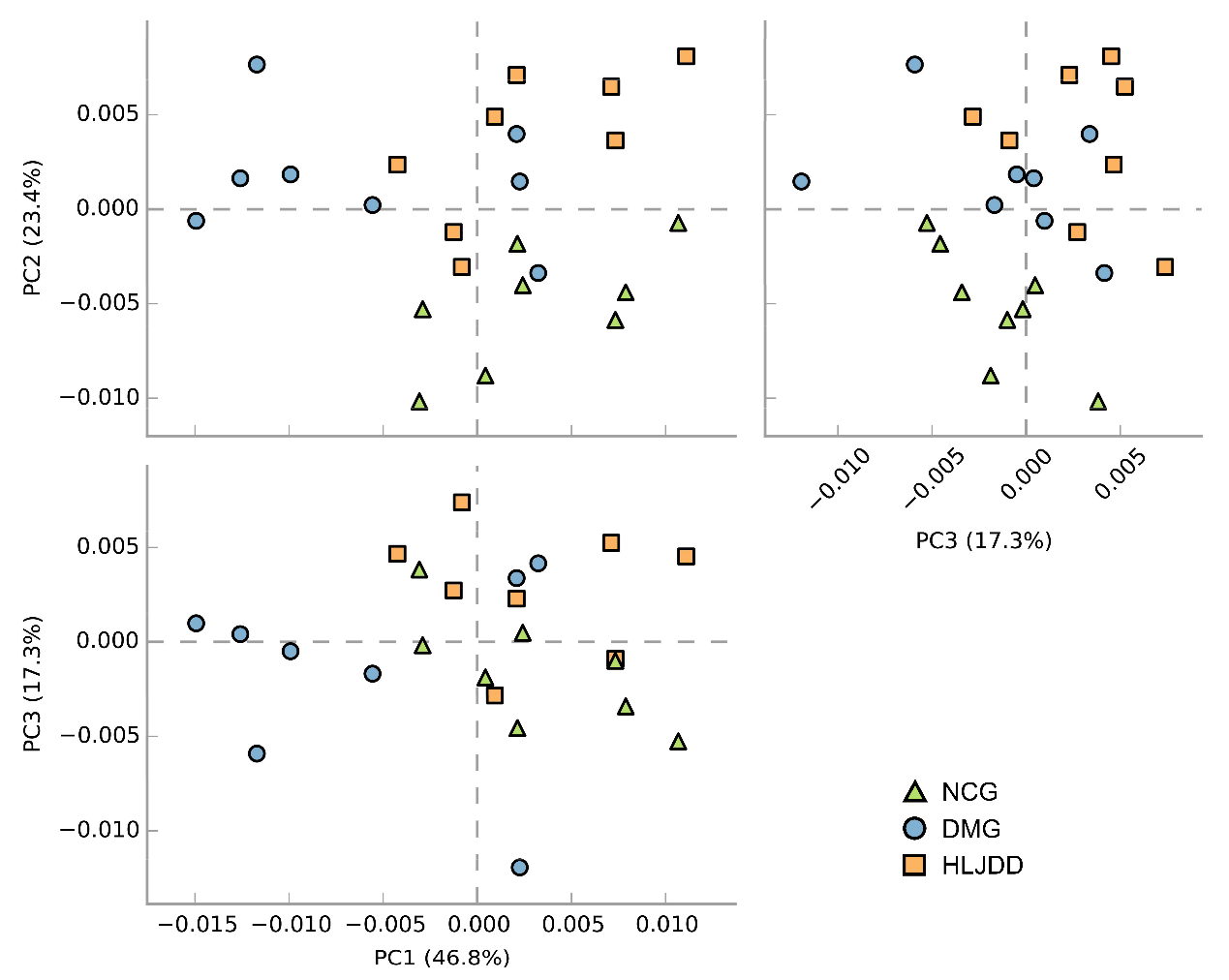


**Supplementary Figure 4.** Principal components analysis based on the PICRUSt analysis.

## Supplementary Tables

**Table S1 Recipes of control and high-fat diet**

| Ingredient | Control diet (CD) | | High-fat diet (HFD) | |
| --- | --- | --- | --- | --- |
|  | g | kcal | g | kcal |
| Casein | 189.58 | 758.32 | 233.06 | 932.24 |
| L-Cystine | 2.84 | 11.36 | 3.50 | 14 |
| Com Starch | 298.59 | 1194.36 | 84.83 | 339.32 |
| Maltodextrin | 33.18 | 132.72 | 116.53 | 466.12 |
| Sucrose | 331.77 | 1327.08 | 201.36 | 805.44 |
| Cellulose | 47.40 | 0 | 58.26 | 0 |
| Soybean Oil | 23.70 | 213.30 | 29.13 | 262.17 |
| Lard | 18.96 | 170.64 | 206.84 | 1861.6 |
| Mineral Mix M1002 | 9.48 | 0 | 11.65 | 0 |
| Dicalcium Phosphate | 12.32 | 0 | 15.15 | 0 |
| Calcium Carbonate | 5.21 | 0 | 6.41 | 0 |
| Potassium Citrate | 15.64 | 0 | 19.23 | 0 |
| Vitamin Mix V1001 | 9.48 | 37.92 | 11.56 | 46.24 |
| Choline Bitartrate | 1.90 | 0 | 2.33 | 0 |
| Food Coloring | 0.047 | 0 | 0.058 | 0 |
| Total | 1000 | 3845.7 | 1000 | 4727.09 |

**Table S2 Effects of HLJDD on organ index of rats**

| Group | NCG | DMG | HLJDD |
| --- | --- | --- | --- |
| liver (mg/g) | 2.59 ± 0.21** | 3.68 ± 0.20 | 3.42 ± 0.31 |
| kidney (mg/g) | 0.57 ± 0.04** | 1.29 ± 0.15 | 1.18 ± 0.12 |
| pancreas (mg/g) | 0.26 ± 0.06** | 0.36 ± 0.07 | 0.25 ± 0.04* |

Results were expressed as mean±SD, n = 8. **p* < 0.01, **p* < 0.05 vs. DMG.
